# Flavour encapsulation: A comparative analysis of relevant techniques, physiochemical characterisation, stability, and food applications

**DOI:** 10.3389/fnut.2023.1019211

**Published:** 2023-03-02

**Authors:** Marcia English, Ogadimma Desmond Okagu, Kristen Stephens, Alex Goertzen, Chibuike C. Udenigwe

**Affiliations:** ^1^Human Nutrition, Saint Francis Xavier University, Antigonish, NS, Canada; ^2^Department of Chemistry and Biomolecular Sciences, Faculty of Science, University of Ottawa, Ottawa, ON, Canada; ^3^Department of Food and Human Nutritional Sciences, University of Manitoba, Winnipeg, MB, Canada; ^4^School of Nutrition Sciences, Faculty of Health Sciences, University of Ottawa, Ottawa, ON, Canada

**Keywords:** flavour, encapsulation, proteins, flavour stability, surface morphology, rheology

## Abstract

Flavour is an important component that impacts the quality and acceptability of new functional foods. However, most flavour substances are low molecular mass volatile compounds, and direct handling and control during processing and storage are made difficult due to susceptibility to evaporation, and poor stability in the presence of air, light, moisture and heat. Encapsulation in the form of micro and nano technology has been used to address this challenge, thereby promoting easier handling during processing and storage. Improved stability is achieved by trapping the active or core flavour substances in matrices that are referred to as wall or carrier materials. The latter serve as physical barriers that protect the flavour substances, and the interactions between carrier materials and flavour substances has been the focus of many studies. Moreover, recent evidence also suggests that enhanced bioavailability of flavour substances and their targeted delivery can be achieved by nanoencapsulation compared to microencapsulation due to smaller particle or droplet sizes. The objective of this paper is to review several relevant aspects of physical–mechanical and physicochemical techniques employed to stabilize flavour substances by encapsulation. A comparative analysis of the physiochemical characterization of encapsulates (particle size, surface morphology and rheology) and the main factors that impact the stability of encapsulated flavour substances will also be presented. Food applications as well as opportunities for future research are also highlighted.

## 1. Introduction

**Figure 1:**
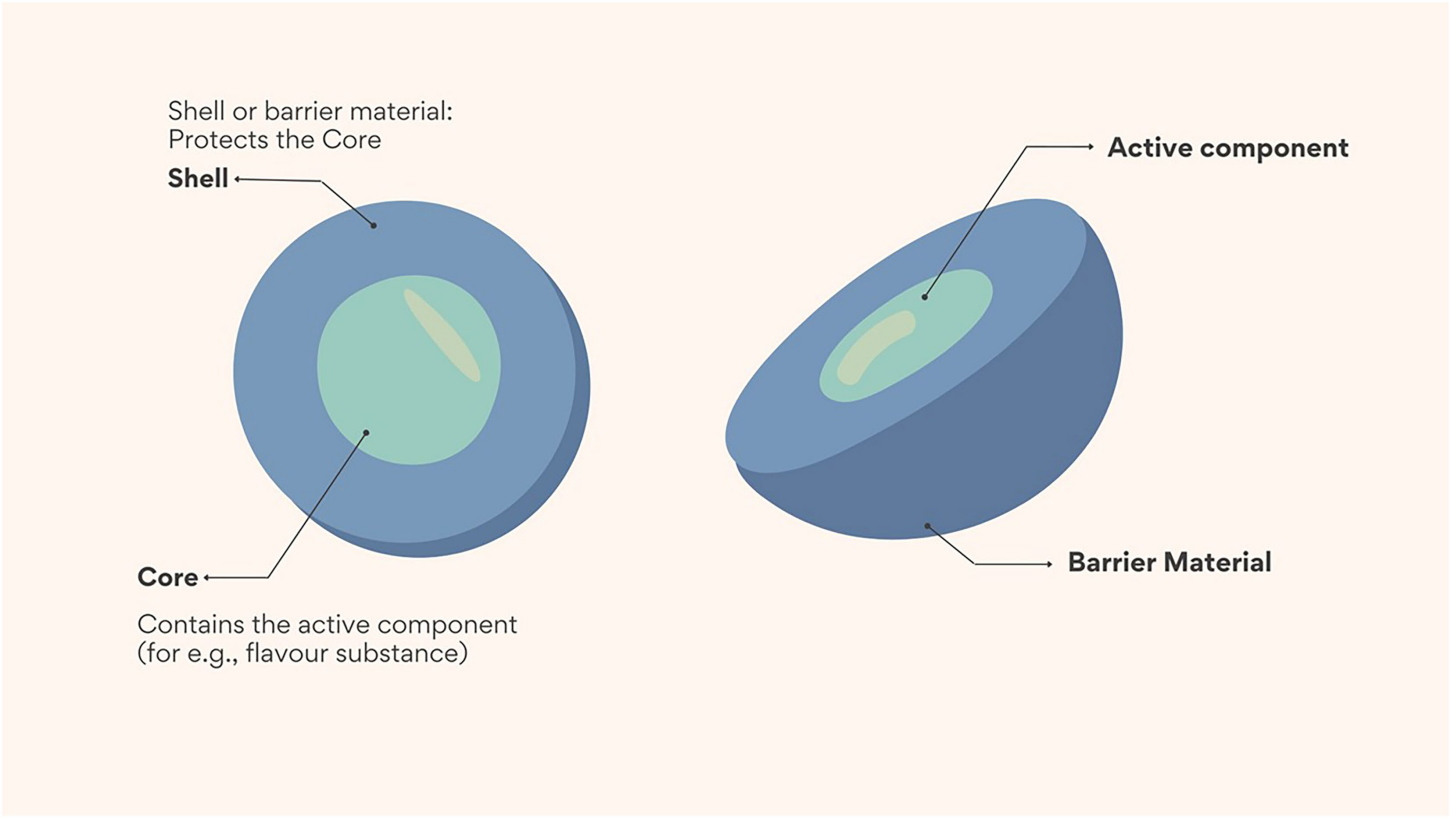
Schematic showing the main structure of a microcapsule. Shell (also referred to as wall or carrier materials) are used as a physical barrier to trap core or active components such as flavour substances.

Flavour is an important component that impacts the quality and acceptability of food (1). The successful development of new functional foods requires the delivery of flavour substances in a manner which does not compromise their stability and quality (2). However, most flavour substances are low molecular mass volatile compounds, and stabilizing them during processing and storage remains a major challenge in some food applications because of their sensitivity to air, heat, and light, susceptibility to evaporation, and poor dispersibility in hydrophilic matrices (3). Encapsulation in the form of macro (> 5,000 μm), micro (1–1,000 μm) and nano (1–100 nm) technology has been used to address this challenge, although micro encapsulation is used more often than nano encapsulation in the food industry (4). During encapsulation, flavour substances are trapped in carrier or wall materials; and the trapped flavour substances are then referred to as core or active ingredients (Figure 1). In this design, the wall material acts as a physical barrier between the sensitive core ingredient and the outer environment which in turn preserves the stability of the flavour substances during food processing and storage (3). However, recent evidence suggests that nano encapsulation technology may be more effective at encapsulating flavour substances and offers improved stability, encapsulation efficiency, and controlled release (3). The improved functionality may be attributed to the smaller particle size and increased surface area achieved with nano encapsulation (5).

The discovery and development of encapsulation in the food industry has had a long history (6). Microencapsulation was first developed by Green and Schleicher in the 1950’s, however, it was Norio Taniguchi who first used the term nanotechnology in 1974 (5, 7). Taniguchi’s definition of nanotechnology referred to the design of materials at the nanoscale, and it was from this concept that nanoencapsulation technologies emerged in the food industry (5, 8). Today, several techniques including spray drying, spray chilling, emulsification, and coacervation have been used to develop micro or nano particles of flavour substances (3, 5, 9). However, successful flavour encapsulation is still challenging because flavour substances can diffuse through the encapsulation matrix and participate in chemical and non-chemical interactions that lead to off-flavours which can have negative effects on food acceptability and ingredient functionality (10). Off-flavour development has been attributed to differences in the properties of wall materials as well as differences in the composition of the interfacial areas compared to the bulk wall materials, which in turn impact barrier properties such as oxygen diffusion, and lead to the instability of flavour substances (10). Thus, many studies have focused on optimizing the physical and chemical properties of wall materials for flavour encapsulation (10). Some of these studies will be highlighted later in this review.

The wall or carrier materials used for encapsulation are based on biodegradable, food-grade, lipids, protein, or polysaccharides (3). However, prior to the mid-1980s encapsulation techniques were primarily developed to protect flavour substances with limited ability to control the release of these ingredients (6). Thus, in addition to their barrier function, the ability of wall materials to allow for the controlled release of flavour substances at specific rates under certain conditions is of interest to food manufacturers (2). This delayed release is an important feature that further protects flavour substances and enhances the quality of food (2). In this regard, nanoencapsulation is believed to be more effective than microencapsulation (3). Importantly, since the blending of different polysaccharides was reported to improve functionality, earlier studies investigated the stability of flavour substances encapsulated in wall materials composed of various polysaccharide blends (11, 12). The properties of these wall materials have also been characterized using electron microscopy and dynamic light scattering technique (13). Moreover, because microencapsulation of flavour substances by spray drying is frequently used in the food industry, many studies have investigated the impact of wall material composition on the functionality of the core materials (14). Recently these authors (1) investigated the dynamic release of the flavour substance (ethyl acetate) trapped in wall materials composed of maltodextrin with varying levels of dextrose equivalent (DE) values and relative humidity values (22, 43, 65, and 75%) (1). Varying the DE value has important effects on the functional (solubility, viscosity, and emulsifying) properties as well as the structural properties of maltodextrin (1). The headspace concentration of ethyl acetate was measured using a gas chromatography mass spectrometry (GC–MS) approach, and maltodextrin was shown to be effective at reducing the diffusion of the ethyl acetate through interactions between the core and wall materials. Optimizing the encapsulation properties of wall materials is still an active area of flavour research today (15).

Designing effective encapsulation systems lies in: (1) understanding the chemical properties of the flavour substances and the carrier materials; (2) choosing the appropriate methods to characterize the encapsulated system; and (3) knowing the processing conditions as well as the end use of the product (16, 17). The current literature contains many in-depth reviews on flavour and nano and micro-encapsulation technologies (2, 18, 19). Rather than presenting a detailed discussion of these technologies, this paper will review several relevant aspects of physical-mechanical and physicochemical techniques employed to stabilize flavour substances by encapsulation. This section will be followed by a comparative analysis of the physiochemical characterization of encapsulated particles (particle size, surface morphology and rheology) which is a novel contribution of this work. The application of the encapsulated particles in different food products, as well as interactions that impact their stability have also been reviewed. Current opportunities for future research have also been highlighted.

## 2. Novel encapsulation techniques used to stabilize flavour substances

The two main types of encapsulation technologies employed for the protection and delivery of flavours and aroma compounds are nano- and micro-encapsulation, give rise to various encapsulation systems such as nano/microcapsules, molecular inclusion complexes, micro/nanoparticles, solid–lipid microparticles, crystalline particles, fibrous films, nanotubes, nanoparticles, conventional or micro/nanoemulsions, and multiple emulsion (3). These encapsulation technologies are important in food systems because they have been used to decrease the volatility, oxidation, evaporation, thermal, photo and chemical degradation of flavour substances, as well as to improve gustatory and olfactory perception (20, 21). Various materials such as proteins, carbohydrates, lipids, gums, inorganics, cyclodextrins and polymeric materials have been employed as wall or carrier materials and have not only enhanced retention and chemical stability of flavour compounds but have also demonstrated controlled and sustained release (3, 22–24). Enhanced bioavailability of flavour and the possibility of their targeted delivery can be achieved by nanoencapsulation compared with microencapsulation due to smaller particles or droplet sizes (25). Importantly, all these carrier materials must be granted GRAS (generally recognized as safe) status if they are to be used in food applications (26).

Various factors influence the type of micro- or nano-encapsulation technique employed such as the structural, physicochemical, and functional properties of the flavour compounds and the carrier materials, their food matrix compatibility, as well as the type or intended use of the final products (26, 27). For example, oxygen sensitive flavour substances have been shown to be more stable in wall materials prepared by emulsion systems (28). Moreover, in these emulsions systems, antioxidants may be used as inhibitors to slow down the degradation of flavour substances in citrus oils for, e.g., citral (29). Citral is an α, β-aldehyde with an intense lemon flavour and aroma, however, it is chemically unstable and degrades over time due to acid-catalysed and oxidative reactions (30). Indeed, researchers (29) have shown that citral degradation was reduced in an oil-in-water nanoemulsion systems (pH 3.0) that incorporated the antioxidants, β-carotene and black tea extracts after 60 days of storage at 25 and 50°C. However, citral degradation occurred more quickly in formulations stored at 50°C further emphasizing that increasing temperature can decrease the viscosity of the phases in the system and result in volatilization of flavour substances (31).

The type of encapsulation technique chosen can also result in encapsulated products of various sizes, shapes, permeability, stability, shell thickness, miscibility, and flavour compound release rates (3, 9, 32). Different encapsulation technologies have also been used to modify the physicochemical properties of the carrier materials and flavour substances to produce micro/nano mononuclear, polynuclear and matrix of spheres, capsules, fibres, or vesicles of interest (27). Some studies have supported the adoption of nanoencapsulation technologies since most biological processes occur in the nano range and because the process has been reported to produce better capsule stability, encapsulation efficiency, and controlled and sustained release (3). However, not all authors agree, and in a recent report microencapsulation technology was reported to be more effective in producing higher encapsulation and loading efficiency, control and sustained release, easier handling, and industrial scalability compared to nanoencapsulation (33). This alternate view indicates that the techno-functional, physicochemical, and biological properties of nano or microencapsulation system could be mainly dependent on the nature of the flavour substances, the carrier materials, the encapsulation technology, and conditions of encapsulation.

**Figure 2:**
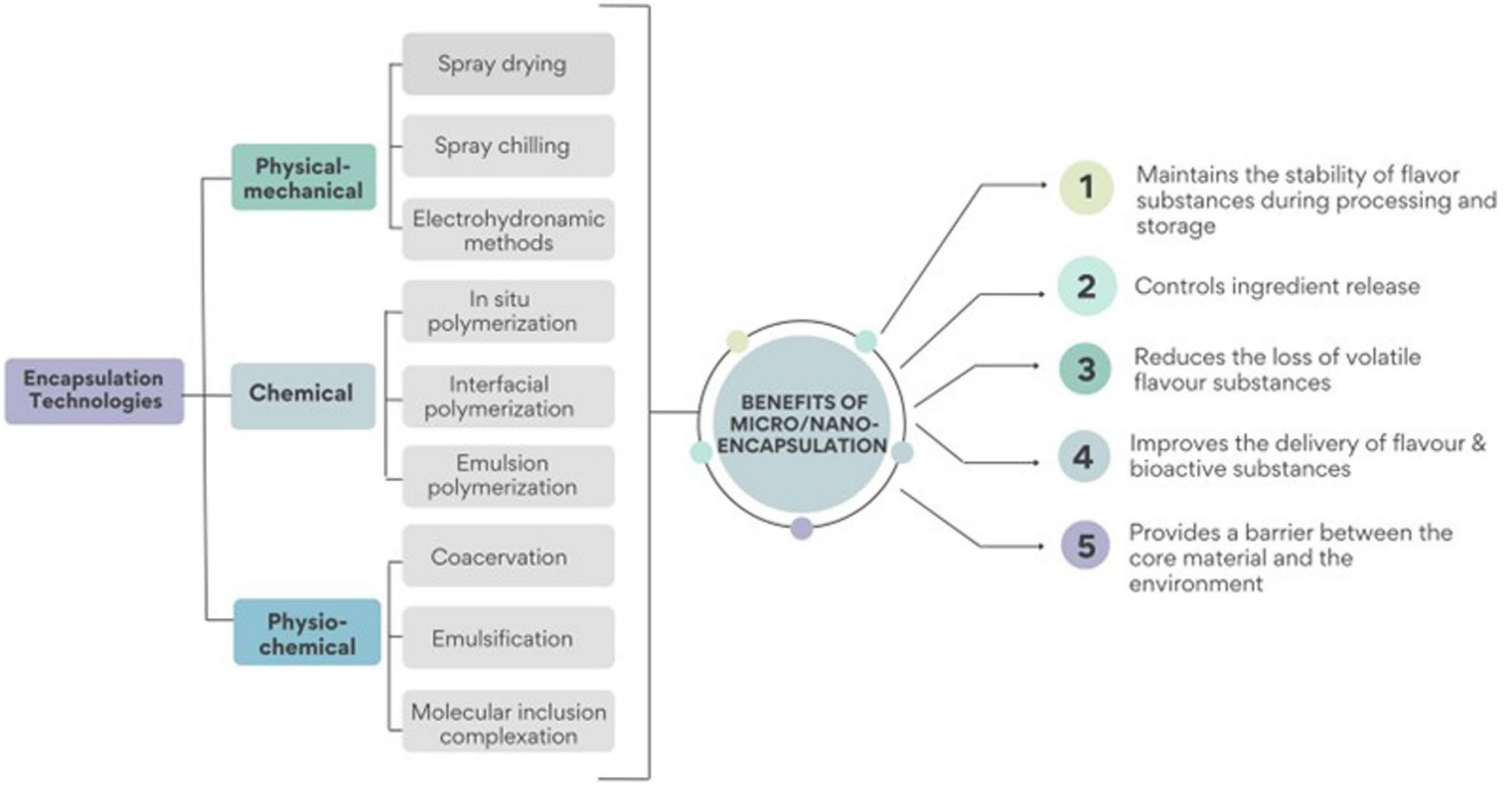
Encapsulation technologies can be classified into three main groups: (1) physical–mechanical methods, (2) chemical methods, and (3) physicochemical methods. The application of these technologies results in improved flavour stability during processing and storage, as well as improved delivery and release of flavour substances among other benefits.

Different encapsulation technologies can also produce micro or nano range flavour-loaded polymeric food substances depending on the processing conditions such as temperature, pH, separation methods, concentration, drying, as well as the nature of the flavour compound and the carrier materials (7). The various encapsulating technologies may be broadly classified into three categories Figure 2: (1) physical–mechanical methods such as extrusion, freeze-drying, spray chilling, spray freeze drying, spray drying and electrohydrodynamic methods; (2) chemical methods including interfacial, emulsion or *in situ* polymerization (34); and (3) physicochemical methods namely coacervation, emulsification and antisolvent precipitation and molecular inclusion complexation (3, 35).

Although many researchers have classified the encapsulation by inclusion complexation as a chemical method (34), the latter may be distinguished from physiochemical encapsulation methods by the nature of the forces that stabilize molecular inclusion complexes (36). More specifically, the walls of the carrier materials formed by physical-mechanical and physicochemical methods are held by non-covalent interactions (ionic, phase separation, hydrophobic and hydrophilic) and therefore are of low strength, biodegradable and applicable in food formulation (34). Conversely, chemical methods form encapsulating walls of high mechanical strength and encapsulation efficiency by covalent interaction and are mostly applicable in the encapsulation of fragrances and oils for textile, insect repellent and footwear applications rather than food (37, 38).

Thus, while physicochemical and physical-mechanical methods can be employed to encapsulate flavours with natural and synthetic encapsulating agents, chemical methods are preferable when working with synthetic delivery shells for effective manipulation of particle sizes, encapsulation and loading efficiency, dispersity, and shapes (39, 40). The major challenges limiting the application of chemical methods in food formulation are their non-biodegradability and biocompatibility, toxicity associated with the use of some reagents such as isocyanate and formaldehyde, and high volume of residual solvents (41, 42). A brief overview of the main physical–mechanical and physiochemical encapsulation methods will be highlighted in the next section.

### 2.1. Physical–mechanical encapsulation methods for flavour substances

#### 2.1.1. Extrusion

Extrusion is a physical-mechanical and gelation technique that is used in the encapsulation of flavour substances (3, 9). Here, an aqueous solution of the wall material is prepared (for, e.g., sodium alginate), and the flavour substances are added, then either a nozzle, a spinning disk or a double capillary is used to force the mixture into a gelling environment (e.g., calcium chloride solution) (3, 43). Due to the low temperature conditions (rarely exceeding 118°C), this technique is useful for the encapsulation of heat degradable flavour substances (44). In addition, extrusion techniques may be classified as electrospinning/electrostatic, jet cutting, co-extrusion/centrifugal, coaxial airflow, dripping/prilling, particle from gas saturated solution, hot–melt extrusion, and melt injection depending on the type of extruder used, the extrusion conditions, the type of extrudate produced (45), as well as the droplets formation mechanisms such as surface tension, frictional, impulse, and gravitational forces (3). The gel droplets formed are converted to capsules by chemical or physical processes such as gelation, heating, and cooling (43, 46). The size of the particles formed after extrusion depends on the nozzle diameter, flow rate of the colloidal dispersion, and the electric potential (47).

However, in comparison to other extrusion techniques, electrostatic extrusion is very effective at producing small particles (~50 μm) (48) albeit outside the acceptable range for nanoparticles (1–200 nm) (48). In this type of extrusion, electrostatic forces are used to disrupt the liquid filament at the top of a needle as well as to create a charged stream of small droplets (49, 50). Indeed, research has been done to investigate the impact of electrode geometry on electrostatic droplet generation and particle size (49). In this study (49), calcium alginate solutions (0.8 and 1.5%, *w*/*v*) were subjected to different charge geometry (electrical field and surface charge intensity) and electrode spacing, and the droplets were exposed to a gelling solution containing 1.5% (*w*/*v*) calcium chloride. The authors observed that when the alginate concentration was decreased from 1.5 to 0.8% the average bead diameter decreased 10–20%. Moreover, reducing electrode distance (4.8–2.5 cm) resulted in smaller particle sizes (from 1,500 to 350 μm diameter) (49). Although the concentration of alginate did not have a significant effect on particle size, the shape of the polymer droplets formed in an electric field has been reported to be affected by changes in viscosity (51). Balanč et al. (51) proposed that extrusion of high-viscose alginate solutions (3 and 4%) resulted in egg-like or drop-like shaped particles. In a similar study, electrostatic extrusion was also used to produce beads made from calcium alginate and sodium alginate and D-limonene (5 and 10%, *w*/*w*) solutions (50). Lević et al. (50) also showed that the size of the flavour droplet was affected by the concentration of the polymer solution and that the application of electrostatic extrusion and ion exchange led to the formation of less spherical and smaller beads compared to those formed without an electrostatic field (50). These results further emphasize the need to optimize processing parameters with the concentration of the polymer or wall materials used for encapsulation, since poor networks may not provide the necessary barrier effects needed to protect the flavour substance (46).

An alternate extrusion technology is melt extrusion which uses a machine known as an extruder (45). Here, raw materials (wall material(s)) are fed into a barrel and heated, the barrel is connected to a thermostat to regulate the temperature of the extrusion process. One or two rotating screws then push the material towards a die or cutter (45). During the melt extrusion process, the addition of plasticizers is advantageous for flavour encapsulation, as they allow for a reduction in the glass transition temperature of the wall material, which in turn makes encapsulation occur at lower temperatures (45, 52). This process protects flavour substances which are added to the plasticized wall material at a later stage in the extrusion process (52). In a recent study, melt extrusion was used to encapsulate orange oil into matrices of octenyl succinate starch (this polymer is used as an emulsifier for flavours) and maltose polymers of varying molecular weight through twin-screw extrusion (53). The study showed that the molecular weight of the matrix, birefringence intensity, moisture content, and gelatinization of the modified starch all affected oil payload (53).

Although extrusion is a simple and cost effective technique at the laboratory-scale and has been shown to increase gel particle shelf life, there are however, some limitations associated with this encapsulation technique (46). For example, it is quite difficult to produce nano-range particles or capsules. Also, large-scale production is costly and only a few carrier materials can be employed in the process since the processing of viscous polymer solutions is challenging (54). Specifically for melt extrusion, the non-reproducibility of data collected after reconstitution of extruded droplets due to its high sensitivity to shear of mixing is a major challenge. Another limitation is related to low oil payload, which usually arises from samples with low oil phase-to-carbohydrate melt viscosity ratios, coalescence of droplet during extrusion, and low shear rates (53, 55, 56).

#### 2.1.2. Spray drying

Spray drying is a rapid, simple, and relatively cost-effective encapsulation technique. This method is the most widely employed encapsulation technique to produce powdered flavour by the solidification of a homogenized colloidal solution of the flavour compound and delivery agent (57, 58). The process involves the atomization of the homogenized solution in the dryer chamber by a spinning wheel or nozzle followed by evaporation of the aqueous solvent when subjected to hot air (temperatures between 150 and 300°C, depending on the material) to produce mainly spherical powdered microparticles, which are collected through a cyclone separator (59, 60). However, the actual temperature exposed to the materials in the atomized particles is less than the temperature of the air due to the latent heat associated with liquid evaporation as well as the high surface-to-volume ratio of particles which allows rapid drying and minimizes thermal damage (59). Prior to spray drying, the solution can be homogenized with ultrasonic emulsification, high-pressure homogenization or microfluidization (3). This method can only be employed when the wall material and flavour substance have high solubility in water and the resulting solution has low viscosity, high emulsifying properties, and ability to form film with air-drying properties (14, 60).

Different materials can be employed as wall materials including polysaccharides (maltodextrin, sodium alginate, chitosan, and carrageenan) and proteins (whey protein isolate and soy protein) (60–62). Accurate selection of the ratio of the flavour substance and the wall material is essential in micro and nanoencapsulation. For instance, a high encapsulation efficiency of 93% was reported after applying spray drying to encapsulate cinnamon essential oil into a wall material comprised of whey protein isolate, maltodextrin, and sodium alginate at an optimum ratio of 70% (carrier/cinnamon essential oil) (63). In another study, the spray drying technique was used to encapsulate Baltic herring oil into rice and whey proteins, and the emulsions formed at pH 6.5 produced a powder that was resistant to oxidation (64). A recent study also employed spray drying microencapsulation technology in the production of gastric resistant citral-loaded soy protein–soy polysaccharide Maillard reaction products for intestinal delivery of citral in chickens (62). The Maillard reaction product formed a better encapsulating agent compared to a physical mixture of soy polysaccharide and soy protein (62). Some researchers (64) also reported that the nature of wall material formed by spray drying affects the oxidative stability of the loaded flavour compound. In their report, fish oil loaded in a combination of pea, soy, sunflower, and whey protein showed better oxidative stability than when whey protein was replaced with maltodextrin (64).

The compatibility of the constituent used as well as the wall material is essential to the stability of the loaded flavour substances especially in preventing cracking (64). However, there are limitations associated with the adsorption of the powder product to the spray dryer leading to considerable loss of product. Also, this technique can lead to the degradation of thermally unstable flavours (65) and flavour loss due to chemical reactions induced by the operating temperatures and the diffusion of the flavour substances through the wall materials (10).

#### 2.1.3. Spray chilling

Spray chilling, which is also known as prilling, spray congealing and spray cooling, is a microencapsulation technique that employs similar operation process as spray drying except that the hot air chamber is replaced with a cooling chamber (66). Here, the atomized homogenized solution of wall material and aroma compound is solidified by passing through a cooling chamber of temperature lower than that of the melting point of the encapsulating agent (Table 1). The temperature of the atomizing air is usually 10°C above the melting point of the lipid used (71). In the spray chilling technique, only lipid material is used as the wall material for flavour substances, and this differentiates it from spray freeze drying (71). The potential applications of spray chilling in the encapsulation of aroma compounds have been reported. For example, 2-acetyl-1-pyrroline zinc chloride complex encapsulated in paraffin (72) and octacosane (73) by spray chilling showed good storage stability and heat-induced controlled release. Due to the reduced level of flavour loss by thermal degradation and evaporation at high temperature, this technique is regarded as a better alternative to spray drying and is useful in the encapsulation of heat-sensitive flavour compounds. A major limitation of spray chilling is the difficulty in controlling particle size (74). Also, only lipid material can be used as the wall material thereby limiting its food applications. In addition, the encapsulated material used must be stable at the temperature necessary for melting the lipid matrix (67, 71).

**Table 1:** Key features of spray drying and spray chilling.

Key features	Spray drying	Spray chilling	References
Steps in the process	1. Preparation of the infeed dispersion (wall material carbohydrates/proteins, plus flavour substance). 2. Atomization of the dispersion. 3. Drying of the atomized particles. 4. Separation of the dried product from air.	1. Adding the flavour • substances to the carrier material (usually lipid). 2. Atomization of molten material. 3. Atomized material meets cool air or liquid N_2_. 4. Solidification of wall material and formation of particles.	(26, 60, 67)
Direction of energy flow	Energy is applied to the droplet, forcing solvent evaporation.	Energy is removed from the droplet, forcing solidification of the wall material.	(67)
Average size of particles	1–150 μm	20–200 μm	(68, 69)
Particle morphology	Spherical and smooth	Rough surfaces and spherical shape	(70)
Advantages	Cost-effective Continuous production Quick process time	Relatively low cost and reduces energy use (no evaporation of water) Use of low temperature in the process Easy to scale up production.	(66)
Disadvantages	Limited number of wall materials are suitable. Adsorption of powder product to spray dryer and can result in product loss.	Encapsulated material must be stable at the temperature necessary for melting the lipid matrix (for, e.g., vegetable oil melting point, 32–42°C). Only lipid material can be used as the wall material thereby limiting its food applications. Insoluble in water due to lipid coating.	(67, 71)

#### 2.1.4. Electrohydrodynamic methods

The two commonly employed electrohydrodynamic encapsulation techniques for flavour compounds are electrospraying and electrospinning (3). Electrospinning is a simple and low-cost microencapsulation technique that, in conjunction with emulsion or coaxial method, could also produce nanoparticles of encapsulated flavour compounds (3). The application of high voltage direct current withdraws polymer solution from a syringe and spins them into fibre, which are received from the collector. The equipment consists of a source of high voltage, a capillary pump, and a collector (3). Electrospinning has been used in the production of nanofibers in the size range of 10 to 100s of nanometres (75). Although electrospraying uses the same apparatus as electrospinning; the only difference is that electrospinning produces nanofibers whereas electrospraying produces nanodroplets. The determining factor is the product of the intrinsic viscosity of the solution and the biopolymer concentration which is known as the Berry’s number (76).

Nanofibers are formed at high concentration when the Berry’s number is above the critical value, but nanodroplets are formed when the Berry’s number of a polymer solution is lower than this value, especially at low concentrations (77). The lengthening and stabilization of the spinneret jet at high polymer concentration leads to the production of nanofibers by electrospinning while the destabilization of the jet from the nozzle containing low polymer solution produces nanoparticles or droplets by electrospraying (78). The technique is recommended for the encapsulation of heat-sensitive flavour compounds because heat is not involved in the encapsulation process, and they produce particles and droplets with very low polydispersity index.

Many proteins can be employed to encapsulate flavour compounds by electrospinning but in electrospraying, some proteins are not compatible with the technique and might require the addition of a surfactant or a plasticizer (79). The complex macromolecular and 3D structures in addition to strong intra and intermolecular forces in proteins have contributed to their limited use in electrospraying (79, 80). Electrospinning and electrospraying techniques were employed in the encapsulation of picrocrocin, safranal and crocin with zein to achieve encapsulation efficiency between 74 and 97%. The products maintained their structural integrity upon exposure to moisture, showed high photostability, and demonstrated the diffusion release mechanism (81).

### 2.2. Physicochemical encapsulation methods for flavour encapsulation

#### 2.2.1. Coacervation

Coacervation is a nano and microencapsulation technique that employs either aqueous or non-aqueous phase separation in polymeric dispersion at a specific condition to produce liquid polymer-rich phase called coacervate (79, 82). The principle involves emulsion formation followed by precipitation of the continuous phase around the droplets of the discontinuous phase (79). Micro-/nano-encapsulation systems are produced through coacervation by first preparing three immiscible phases, followed by the adsorption of the encapsulating agent around the flavour substance before solidification to form micro or nano capsules (52). The solidification and size reduction step in coacervation can be challenging for heat-sensitive flavour compounds as it involves desolvation, heating or cross-linking, which might influence the structural integrity and functionality of the aroma compound (52). The materials employed during coacervation are the solvent, the flavour substance and the wall material (s). This method can produce either simple or complex coacervates depending on the reaction condition and number of solutes in the system (83).

Simple coacervates are obtained by salting out with electrolytes or by desolvation using water miscible nonsolvent and contains only one colloidal solute while complex coacervates are produced through polyelectrolyte complexation methods involving more than one colloidal solute. Complex coacervation are more applicable in food flavours than simple coacervate as they possess better encapsulation functionality resulting from their multiple encapsulating walls (52). Using this technique, hydrophobic and hydrophilic flavour compounds can be encapsulated by aqueous and non-aqueous phase separation, respectively (82, 83). The coacervation method has been used in the encapsulation of flavour substances for food applications using encapsulating agents derived from proteins and carbohydrates. For instance, a stable vanillin-loaded casein–sodium alginate complex coacervate formed through polyelectrolyte complexation demonstrated sustained release of the flavour compound (84). Also, a comparative study using gelatin–chia mucilage and gelatin–Arabic gum as the encapsulating agent for oregano essential oil registered an encapsulation efficiency above 90% with the former forming a better complex coacervate (85).

In another study, simple coacervation technique was employed in the encapsulation of holy basil essential oil into gelatin. Response surface methodology was used to optimize the encapsulation conditions and an encapsulation efficiency above 90% was reported at the optimum condition (86). A thermally stable coacervate was fabricated from canola protein isolate and chitosan for potential encapsulation of nutraceutical compounds including flavour and aromas (57). The optimum condition for high yield coacervates was a canola protein isolate-chitosan mass ratio of 16 and pH 5.8–6.2. The thermal stability of the coacervate increased further on cross-linkage of the protein with transglutaminase (57). Although coacervation techniques have demonstrated interesting applications in the formulation of functional aroma-enhanced food products especially heat-sensitive ones that could be degraded by other methods such as spray drying, there are some challenging limitations (3). Notably, it is difficult to achieve consistent quality control of the encapsulation process since a change in one of the many encapsulation variables (pH, ionic strength, molecular weight, solvent, and nature of flavour compound) can lead to different sizes and particle accumulation. This could result in high production costs and capsules of varying stability in different solvent environment (83).

#### 2.2.2. Formation of molecular inclusion complexes with cyclodextrins

Molecular inclusion complexes formed by modified starch molecules, cyclodextrins, have been investigated as potential delivery systems for microencapsulation of flavour and aroma compounds. Structurally, cyclodextrin has a hydrophobic core for ligand entrapment and hydrophilic surface for interaction with aqueous environment. The binding of the bioactive compounds through hydrophobic interaction in the hollow truncated cone-like nano-sized cavity of cyclodextrin enhances the encapsulation of biomolecules (3, 74).

Flavour compound–loaded molecular inclusion complexes of cyclodextrin have shown enhanced stability, protection, and controlled and sustained release (87). Molecular inclusion complexes of γ-cyclodextrin with watermelon flavour promoted the sustained release of the flavour but modified the aroma composition during inclusion and release (88). The molecular structure of the watermelon flavour affected the inclusion and release process. Flavour alcohols derived from watermelon had higher inclusion efficiency than the aldehyde and ester counterparts, but the most hydrophobic aromas showed lower release ratios due to greater hydrophobic interactions in the cyclodextrin cavity (88).

In another study, the authors prepared amylose flavour-based molecular inclusion microencapsulation complexes of improved thermal stability (89). The encapsulation efficiency increased with decrease in chain length of the flavour compounds, and aromatic naphthol flavour showed lower encapsulation efficiency compared to some linear alcohols (89). The physicochemical properties of molecular inclusion complexes can be enhanced by the application of other encapsulation techniques. For example, when molecular inclusion is combined with electrohydrodynamic technique electrospun nanofibers of high thermal stability, antioxidant and antibacterial properties, sustained release, and improved shelf life have also been reported (90).

## 3. Physiochemical characterization of encapsulated flavour substances

The main physical-mechanical and physiochemical approaches used to encapsulate flavour substances were highlighted previously. However, an important component of flavour encapsulated systems is their physiochemical properties (17). Among the physiochemical properties of interest, their particle size and surface morphology have been previously studied, however, fewer studies have focused on the rheological properties (15, 91, 92). However, interest in understanding these properties is still growing because of their impact on the stability and functionality of the encapsulated flavour substances (68, 93). Some of these physiochemical properties and their effects on the stability of encapsulated flavour substances will be reviewed below.

### 3.1. Particle size of encapsulated flavour substances

The size of micro/nanoparticles formed as well as conventional or micro/nanoemulsions and their distribution (polydispersity) are important properties that determine the functionality of encapsulated flavour substances (92, 94). According to the observations obtained from the literature, the average diameter of particles for spray-dried powders and spray-chilled flavour substances are reported to be in the 1–150 μm range and 20–200 μm range, respectively (68, 69). However, strawberry flavour encapsulated materials by spray drying using different compositions of wall materials (16.12% modified starch, 9.76% gum Arabic 4.12% soluble fibre, and 1% β-cyclodextrin) generated microparticles with a wide range of diameters ranging from 0.32 to 69.25 μm (15). This variation was attributed to the inlet temperature (130–190°C), air velocity, and the size of the oil droplets (15). In general, particles with a smaller diameter will contribute to tighter packing and lead to an increase in bulk density (i.e., the amount of material by weight that will fit into a container of a specific volume). The bulk density is also a measure of the flow properties of the micro/nanoparticles (95).

One of the main methods used to characterize particle size and polydispersity is Dynamic Light Scattering (DLS), which is a technique based on the Brownian motion of dispersed particles (96, 97). In the case of micro/nanoemulsions, the particle size can also be determined by values such as the mean particle radius, surface weighted average or volume weighted average (25). In these emulsion-based systems, polydispersity gives an indication of the aggregation state of the encapsulated system (17). High polydispersity indicates the presence of aggregates, that may destabilize the encapsulation systems (17).

### 3.2. Morphological characteristics of encapsulated flavour substances

Although many encapsulation technologies have been optimized to obtain particle sizes in the acceptable micro/nanoencapsulation ranges, many researches are also interested in knowing the impact of encapsulation on the morphology of the obtained particles (69, 98). Morphology includes the internal and as well as the external structure of micro/nanoparticles and is usually reported using scanning electron microscopy, transmission electron microscopy or atomic force microscopy techniques (99). Different parameters including surface tension between fluids, stirring rates, temperature, as well as viscosity, can impact the morphological properties, the size distribution, as well as the stability of the encapsulated micro/nanoparticles and conventional or micro/nanoemulsions formed (17, 100, 101).

The type of encapsulation technique has also been reported to impact the morphology of micro/nanoparticles (68). Many of the examples in the literature refer to spray-drying since this is the main process used to encapsulate flavour substances (15, 95). For example, durian flavour from a South-East-Asian fruit was encapsulated using spray drying and freeze drying and three different types of wall materials (maltodextrin from corn, wheat and potato) (69). The spray-dried products were reported as small (1–15 μm in diameter) and spherical in shape, whereas the freeze-dried products were described as larger (up to 300 μm) and irregular-shaped. Spherical, smooth and homogenous morphology has also been reported for strawberry flavour encapsulated in spray-dried microparticles (15). In spray dried microparticles the use of high inlet temperatures (~ 150–180°C; with ΔT, difference in inlet and exit air temperatures, ~ 120°C) is one of the main factors that contribute to uniform and smooth surfaces (95, 102). Moreover, less shrinkage and cracks were observed in microparticles, when drying occurred at higher temperatures (150–180°C) (101).

Conversely, roughness may develop in spray dried microparticles when film formation during the drying process slows down (101). Rough microparticles are undesirable because they have a greater contact surface (compared to smooth microparticles) and are more susceptible to oxidation reactions and thus, less stable (98, 103). Roughness can also affect the flow properties of microparticles (due to changes in particle aggregation) which further emphasizes why microparticles with smooth and uniform surfaces are preferred (98, 103).

Similar to spray drying, when flavour substances were encapsulated by spray chilling, spherical microparticle have been reported. However, the microparticles were also described as dense and free flowing (since no solvent evaporation is involved) (67, 68). Importantly, since only lipids are used as the wall materials in spray chilling, the morphology of microparticles produced by this technique will be similar to the lipid matrix (68). In contrast, different morphologies were reported for the encapsulation of fruity flavour substances, ethyl butyrate and hexanal in inclusion complexes formed with beta and gamma-cyclodextrin (β-CD and γ-CD) (104). In this study, scanning electron microscopy revealed CD complexes with columnar shapes and smooth and compact surface texture (104). These findings further suggest that the method used for encapsulation can also impact the morphology of the encapsulated products.

### 3.3. Rheological characteristics of encapsulated flavour substances

Rheology is the science used to describe and evaluate the flow and deformation behaviour of matter or materials (105). Rheological measurements have been used to provide valuable information on the viscosity, network structure and molecular interactions of food systems (106–108). Most of the rheological studies on flavour encapsulation in the literature have focused on evaluating the shear viscosity of emulsions (prior to drying) or the viscoelastic properties of wall materials and how these properties impact the stability or the release of flavour substances from different wall or matrix materials (109, 110). However, some authors have investigated the impact of various properties of wall and core materials (including their rheological properties and surface tension) on the morphology of the micro/nanocapsules formed.

For example, *Alyssum homolocarpum* seed gum (AHSG; 0.5%, *w*/*w*) and D-limonene (10–30%) emulsions were electrosprayed to form nanocapsules (110). Generally, it has been observed that hydrocolloids with high molecular weight and thus increased viscosity when hydrocolloid concentration was increased, resulted in the formation of micro or nanocapsules in the electrospraying process (111). However, increased surface tension of the emulsion can hinder the electrospinning process and lead to the formation of heterogenous aggregated particles (111). Although these authors had determined an optimum viscosity to allow for the electrospinning of *Alyssum homolocarpum* seed gum, the addition of the hydrophobic D-limonene flavour, resulted in a decrease in the surface tension of the gum dispersion which also facilitated the formation of more homogenous and compact particles (110). These findings highlight how the rheological properties (viscosity) of wall materials as well as other physiological properties (surface tension) can impact the composition and the morphology of nanocapsules.

Using another approach, (112) encapsulated ginger essential oil by coacervation and used whey protein isolate and gum Arabic as wall materials. Rheological measurements (creep and recovery behaviour) were used to describe the viscoelasticity of coacervates when ginger essential oils were encapsulated with whey protein isolate and gum. Overall, the addition of the oil containing the ginger flavour caused a decrease in internal viscosity (*η*_1_) and a lower residual viscosity indicating a lower resistance to flow. The addition of the oil containing the flavour substance also caused a decrease in the elastic strength of the coacervate network structure (112).

In a comparative study, flavour substances in garlic essential oils were encapsulated using two different techniques molecular inclusion and complex coacervation and wall materials (β-cyclodextrin and a blend of soy protein isolate and chitosan) (113). The rheological properties of the dispersions with and without the flavour substances were also evaluated using frequency sweep tests and flow behaviour. The dispersions of microparticles that contained the flavour substances registered higher values for storage modulus (G′) and loss modulus (G″) and more compact network structures (113). The findings from this study highlight the importance of enhanced intermolecular bonding and their role in improving microparticle network structures. Such structures are desirable features of microparticles and make them more resistant to structural disintegration of the network chain (113).

## 4. Stability of encapsulated flavour substances

The stability of the micro/nanoparticles as well as conventional or micro/nanoemulsions techniques is strongly correlated with the types of encapsulation method, the size of the flavour encapsulated system as well as the processing conditions (17). In order to be beneficial, flavour encapsulation system, must remain stable under different environmental conditions including temperature fluctuations, light, chemical and mechanical forces (59). Having cracks and collapses in the encapsulated walls is also undesirable, since this can compromise the stability of the flavour substance (core) (98). Thus, the design of effective encapsulation technologies requires knowledge of the chemical properties of the flavour substances, the food matrix, and the characteristics of the wall materials (98).

### 4.1. Impact of interactions between wall and core materials on the stability of encapsulated flavour substances

Two of the main performance criteria of flavour encapsulation is to retain the flavour substances during encapsulation and also to ensure the flavour substance is stable after encapsulation (21). However, when electrostatic extrusion was used to trap peppermint essential oil into calcium-alginate particles, Fourier transform infrared spectroscopy (FTIR) analysis revealed no evidence of chemical interactions (114). Table 2 shows a summary of some of the different techniques that were used to encapsulate various flavour substances. Overall, the encapsulated flavour substances exhibited improved thermal stability and attenuated total reflectance Fourier transform infrared spectroscopy (ATR-FTIR) studies did not reveal any convincing evidence of interactions between the wall materials and the different core materials (flavour substances) (110, 113, 115–118). However, stability can be compromised if any interactions occur between the wall and the core materials. Indeed, (119) have cautioned that physical or physicochemical interactions that occur between the core and the wall materials, (through hydrogen bonding) can form insoluble complexes which in turn can negatively impact flavour retention and stability.

**Table 2:** Physio-mechanical and physiochemical encapsulation methods and representative studies that highlight the interactions between core and wall material and the stability of encapsulated flavours.

	Encapsulation methods	Core/wall material(s)	Interaction between core/wall material(s)	Stability of encapsulated substances	References
Physio-mechanical methods	Extrusion (electrostatic)	Peppermint essential oil/calcium alginate	Fourier transform infrared (FTIR) spectroscopy showed no strong evidence of chemical interactions between peppermint essential oil compounds and wall materials.	Thermal analyses showed that the encapsulated essential oil was released at 150°C and in a more controllable manner compared to free peppermint essential oil. The authors proposed that encapsulated peppermint essential oil may be more suitable for food applications that require high temperatures (e.g., baking).	(114)
	Freeze-drying	Limonene/gum Arabic, sucrose and gelatin	Sucrose and gum Arabic were suitable for encapsulating limonene.	Wall materials with gelatin collapsed during freeze-drying.	(115)
	Spray drying	Polymethoxyflavone (PMF) loaded citrus oil/Maltodextrin	Microcapsules with a high content citrus oil and PMF were formed. However, the appearance of characteristic peaks of citrus oil from ATR-FTIR^1^ studies suggests microcapsule breakage occurred.	Citrus oil microcapsule powder was readily re-dissolved back into the water, and all of them remained stable for at least 24 h. However, there were some differences in the droplet size and microstructure of the reconstituted emulsion.	(116)
	Spray freeze-drying	Vanillin/β-cyclodextrin and whey protein isolate (WPI)	FTIR spectroscopy showed no interaction between the core and wall material.	Thermal stability of the microencapsulated samples and wall materials were analysed using Thermogravimetric analysis (TGA). Spray–freeze-dried vanillin + WPI sample exhibited better thermal stability than spray dried and freeze-dried microencapsulated samples.	(117)
	Electrospraying	D-limonene/ *Alyssum homolocarpum* seed gum (AHSG)	ATR-FTIR^1^ revealed that encapsulation had only minor effects on AHSG structure, confirming that no significant interaction had occurred between the encapsulated flavour and the wall material.	Thermal stability of the core, wall materials and the nanocapsules formed were evaluated by Differential Scanning Calorimetry (DSC).	(110)
Physiochemical methods	Coacervation	Peppermint oil/gelatin and high methyl pectin (cross-linked with tannic acid)	ATR-FTIR showed secondary structure alteration of gelatin in coacervates suggesting the interaction of gelatin and tannic acid.	Peppermint oil revealed a total degradation at 175°C. However, peppermint oil microcapsules exhibited improved thermal stability up to 275°C. Tannic acid-crosslinked coacervates improved the thermal properties of peppermint oil.	(118)
	Molecular inclusion complexes with cyclodextrin	Garlic essential oil (GEO)/β-cyclodextrin	ATR-FTIR was used to investigate possible interactions between wall and GEO. No new chemical bonds on functional groups of wall materials. Encapsulation had no effect on the chemical structure of the flavour substances	Improved thermal stability of GEO flavour substance observed when using β-cyclodextrin as the wall material.	(113)

1 Attenuated total reflectance-Fourier transform infrared spectroscopy (ATR-FTIR).

In another study, the impact of different wall materials on the stability of flavour substances was investigated (117). Here, whey protein isolate, and β-cyclodextrin and combinations of these two components were used as wall materials when vanillin was encapsulated by spray drying, spray-freeze-drying and freeze-drying. FTIR spectroscopy showed no change in the functional groups (aldehyde and ether) which were used to identify the flavour substance in vanillin as well as in the encapsulated samples. However, the combination of wall materials decreased the encapsulation efficiency. In contrast, vanillin encapsulated with whey protein isolate by spray-freeze drying technique exhibited improved thermal stability (460°C), compared to the thermal properties of vanillin encapsulated with β-cyclodextrin (400°C). These findings suggest that the whey protein isolate may be better than the glucose-based β-cyclodextrin wall material at protecting the vanillin flavour substance.

### 4.2. Interactions between encapsulated flavour substances and food matrix components

The food matrix composition can also have an impact on the stability of encapsulated flavour substances. Among food macromolecules, proteins have the most diverse binding behaviour with flavour substances due to the broad variation in their protein structures. Structural protein variation can be attributed to varying amino acid side chains, terminals, and hydrophobic pockets (120). Furthermore, variation in the stereochemistry of flavour compounds is also an important component that impacts protein–flavour interactions (120). Many of these interactions can be classified as reversible (such as hydrophobic interactions, hydrogen bonds, ionic bonds/electrostatic linkages, and van der Waals forces) or irreversible, such as covalent linkages (121). In addition, hydrophobic interactions occur in the interior hydrophobic regions of protein structures and are typically formed with flavour compounds such as ketones, aldehydes, alcohols, and esters. Conversely, hydrogen bonds commonly involve-OH, –COOH, –NH and –SH protein groups and aliphatic alcohols, lactone, and volatile acid flavour compounds (121). Ionic bonds are also commonly formed by electrostatic interactions between anionic and cationic groups of proteins and charged flavour compounds. However, Van der Waals forces are commonly formed between proteins and non-polar flavour compounds. In addition, covalent linkages commonly involve –SH and –NH₂ protein groups and flavour compounds such as aldehydes, and sulphur-containing flavour compounds (120). Flavour substances containing carbonyl groups (for, e.g., aldehydes and ketones) which can react with the amino groups of proteins and result in flavour loss (122).

The two main microencapsulation techniques that utilize proteins include spray drying and coacervation (65). For example, vanillin flavour encapsulated with whey protein isolate using spray-freeze dried samples showed improved thermal stability when compared to spray dried and freeze-dried samples (117). Although carbohydrates provide good oxidative stability to some essential oils, carbohydrates exhibit poor interfacial properties (123). However, milk proteins with their high amphiphilic properties can improve the stability of hydrophobic core materials (123). Thus, it is sometimes beneficial to combine proteins and carbohydrates to stabilize flavour substances. In addition, these authors (124) investigated the binding of unheated and heated whey protein isolate to the flavour compounds 2-nanonone, 1-nonanal, and trans-2-nonenal at pH 4.0, 7.2 and 8.0. Increased binding of the flavour compounds with the native WPI was observed with increased protein concentration. Less protonation of amino groups and other basic amino acid residues as well as conformational changes were proposed to be responsible for these observations (124, 125). In addition, previous studies have shown that the type of extraction can impact the interaction of proteins with flavour substances. For example, canola proteins that were extracted in salt solutions registered higher binding affinities to aldehydes compared to similar proteins that were exposed to salt solutions (126). In addition, except for 2-octanone, other ketone flavour substances (2-hexanone and 2-heptanone) showed higher binding affinities to salt-extracted canola and pea protein compared to the alkaline-extracted proteins. In this study it was proposed that changes in the protein conformation and solubility were responsible for the differences observed in protein–flavour interactions (126).

The encapsulation of flavour substances by coacervation has included proteins from both animal and plant sources (2, 65). Consider as an example the coacervation between soybean protein isolate and gum Arabic which was used for the encapsulation of sweet orange oil (65, 127). In this work, the impact of pH and salt concentration effectiveness of coacervation was also evaluated. At pH 4.0, the highest degree of coacervation was observed, whereas when NaCl concentration was increased to 1 mol/l or higher, coacervation was decreased. The authors proposed that the increase in sodium ion concentration decreased the electrostatic interactions between the polymers (or screened the charges of the biopolymers) which suppressed the formation of coacervates (123).

In food systems, carbohydrate matrices display a diverse capacity for flavour interactions depending on the processing or storage conditions and the flavour molecules (125). The main effects of polysaccharides are related to changes in viscosity and physical entrapment (128). Starch, the major polysaccharide component of food, is made up of two polymers, the linear amylose, and the branched amylopectin (129). The interaction of starch with flavour compounds has been previously described as adsorption and physical entrapment processes that form inclusion complexes (ICs) (129, 130). The formation of ICs is promoted in the presence of flavour compounds because amylose adopts a helical conformation, and its outer surface is hydrophilic whereas the inner surface is hydrophobic. The hydrophobic inner helical channel allows for the stabilization of the ICs by hydrophobic forces (130). Importantly, the formation of starch-flavour ICs is a reversible process, therefore making them potential flavour carriers and ingredients for flavour encapsulation (131).

Simple sugars (such as sucrose) or sweeteners bind easily to water molecules, and thus have a low binding affinity to flavour compounds, which leaves flavour substances to be concentrated in any remaining water, favouring their release (132). These authors (132) further emphasized that the hydration of sucrose (which depends on its molarity) is the rate-limiting factor that determines initial flavour release. Sugars in a food matrix also influence the gelation properties of polysaccharides, which in turn affects aroma release. Biphasic gels of gelatin and starch were also used to study the effect of different sucrose concentrations on aroma release *in vitro* and *in vivo* (132). The authors observed that less hydrophobic compounds (e.g., ethyl ethanoate and hexanal) with more affinity for gelatin were unaffected by sucrose concentrations. Conversely, the addition of sucrose affected flavour compounds that both had higher affinity for the starch phase and had different functional groups (including esters and linear aldehydes), further emphasizing that additional carrier compounds can be selected or introduced to modulate binding and/or release (131).

In addition to their impact on the appearance, and the texture of foods, lipids can bind to flavour substances and by this interaction influence the flavour perception of foods (131). In lipid-containing food systems, the affinity of flavour molecules to the lipid phase depends on its chemical composition, chain length, and the degree of saturation (133). Most flavour compounds are lipophilic (125), and this lipophilicity increases with molecular weight within a homologous series. Importantly, the chain length of the fatty acid determines the polarity of lipids, and in some cases, flavour compounds have higher affinity for lipids with longer chain fatty acids (134). These authors (135) used static headspace chromatography to study the release of limonene and trans-2-hexanal from gum Arabic and propylene glycol alginate solutions. The non-polar limonene demonstrated higher retention compared to the more polar trans-2-hexanal. The preferred entrapment of hydrophobic volatile components was explained by the higher solubility of polar compounds in water, which resulted in higher diffusivity through the matrix compared to the non-polar compounds (135). Furthermore, (136) used 2-heptanone, ethyl butanoate, and ethyl hexanoate to demonstrate significant decreases in volatile release and odour intensities with the increasing addition of fat. Taken together, these observations highlight that interactions with macromolecules (proteins, carbohydrates or lipids) in the food matrix can influence the diffusivity and release of flavour substances.

## 5. Applications of encapsulated flavour substances in the food industry

In the food sector, there has been increased interest in developing functional foods. Foods or food components that have been demonstrated to provide a health benefit beyond meeting basic nutrition may be described as functional foods (137). However, some of the functional ingredients in foods (including flavour substances) are volatile, and are unstable during processing and storage conditions, and thus require the use of novel approaches to ensure their thermal and oxidative stability in different food matrices (138, 139). Flavour substances also play important roles in improving the consumer acceptability of functional foods which in turn promotes their consumption (140, 141). Encapsulated flavour substances are used in a variety of foods including snacks, beverages, and dairy products (141, 142).

In beverages, flavour substances are used in the form of micro or nanoemulsions (141). Orange, lemon-lime and cola are the main flavours of non-alcoholic beverages. Many flavour substances are hydrophobic and before being used they are suspended in an aqueous medium to form a colloidal dispersion (141). The flavour substances in the colloidal dispersions are then encapsulated in micro or nanoemulsions before they are used in the formulation of beverages. Flavoured oils (including orange, lemon, and mint) are used frequently in the food and beverage industry. Furthermore, the composition of these oils will depend on their origin and method of extraction and processing (141). Encapsulated flavour substances in essential oils have been used in spices (cinnamaldehyde in cinnamon), and for the preservation (limonene) of some foods (strawberries) (143, 144).

Production of fermented dairy products (cheese, yogurt and kefir) is one of the main applications for encapsulated flavour substances (142). For example, *Melissa officinalis* essential oil was encapsulated through ultrasonication by using different ratios of whey protein isolate/sodium caseinate as wall materials. Flavoured yoghurt samples were generated by adding the microcapsules to the yogurt formulation and the samples were evaluated at different time points during 3 weeks of storage (145). The sensory evaluation data showed that adding microcapsules to yoghurt samples did not significantly influence the overall acceptability compared to the plain unflavoured samples. In another dairy application, peppermint essential oil was encapsulated in carnauba wax, and the latter was incorporated into ice cream. The use of peppermint oil also imparted a peppermint flavour to the ice cream (114). In related sensory evaluation studies, trained panellists also rated the overall liking of the peppermint ice cream samples above 7 on a scale of 1–10 compared to overall liking scores of 8 for the control which indicates that the strategy of using carnauba wax could be improved upon. Thus, the overall acceptability may also depend on the food product that the encapsulated flavour substance it is applied to.

One of the hallmarks of a successful encapsulation strategy in food systems is one that establishes the stability and controlled/sustained release of the flavour substance. In spite of the successful application of encapsulation in some food products there have been concerns about the possible toxicity of some micro and nano-encapsulation delivery systems (146, 147). Recent reports also advocate for new labelling policies that address some of these concerns (70). However, improved communications between the agri-food industries and consumers are key to increasing consumer awareness and public acceptance of foods with flavour substances modified by encapsulation (147).

## 6. Conclusions and future perspectives

The structural diversity of flavour substances plays a major role in their interaction with the food matrix and subsequent release when food is consumed. As discussed in this review, interactions between flavour substances and the food matrix as well as interactions between flavour substances and wall materials can impact the stability of encapsulated flavour substances. Due to their volatility and low stability when heated, flavour substances may be preserved by using a wide range of physical-mechanical, chemical, and physicochemical methods. The choice of encapsulation method used depends on several factors, especially the chemical properties of the flavour substances and the carrier materials as well as the intended food application. Furthermore, characterization of the physiochemical properties of the flavour substances can provide valuable information about the stability and functionality of the encapsulated flavour substances. Nevertheless, encapsulation systems must be designed in such a way that they not only bind the flavour substances and avoid their release prior to their intended use, but also achieve favourable release profiles to improve their overall acceptability once the product is consumed.

Future studies should further evaluate the favourable controlled release of flavour substances to enhance the application of the encapsulated flavour substances in different food systems. In addition, more work should be done to further investigate the food matrix effect, especially the role of traditional (e.g., fermentation and germination) as well as emerging processing technologies (e.g., pulsed electric field, ultrasound and cold plasma) in altering the food matrix to enhance the binding of flavour substances within the product and their release in the mouth when needed. However, this may be challenging since foods are complex systems and it may be difficult to isolate the specific effect of food molecules (e.g., proteins, lipids and polysaccharides) on the interaction and release of flavour substances. In terms of physiochemical characterization, much of the current literature is focused on evaluating the thermal stability of encapsulated flavour substances, however, in cases where encapsulation efficiency is not optimum, more work should be done to better characterize the oxidative stability of the encapsulated flavour substances. Addressing these knowledge gaps will create more opportunities for the application of encapsulated flavours in new and existing functional food products.

## Author contributions

ME: conceptualization, writing, reviewing, editing, supervision and project administration. OO, KS, and AG: writing and reviewing. CU: conceptualization, writing, reviewing, and editing and supervision. All authors contributed to the article and approved the submitted version.

## Funding

The research programs of ME and CU are supported by the Natural Sciences and Engineering Research Council of Canada Discovery Grants RGPIN-2021-03195 (ME) and RGPIN-2018-06839 (CU), respectively. KS is a recipient of the Nova Scotia Graduate Scholarship, and OO is a recipient of the University of Ottawa International Doctoral Scholarship. ME and CU were also supported by the W. F. James Chair Scholar Program at St Francis Xavier University (ME) and the University Research Chairs Program of the University of Ottawa (CU).

## Conflict of interest

The authors declare that the research was conducted in the absence of any commercial or financial relationships that could be construed as a potential conflict of interest.

## Publisher’s note

All claims expressed in this article are solely those of the authors and do not necessarily represent those of their affiliated organizations, or those of the publisher, the editors and the reviewers. Any product that may be evaluated in this article, or claim that may be made by its manufacturer, is not guaranteed or endorsed by the publisher.
